# Online Recruitment: Feasibility, Cost, and Representativeness in a Study of Postpartum Women

**DOI:** 10.2196/jmir.5745

**Published:** 2017-03-08

**Authors:** Liana S Leach, Peter Butterworth, Carmel Poyser, Philip J Batterham, Louise M Farrer

**Affiliations:** ^1^ National Centre for Epidemiology and Population Health Research School of Population Health The Australian National University Canberra Australia; ^2^ Centre for Mental Health Melbourne School of Population and Global Health University of Melbourne Melbourne Australia; ^3^ Melbourne Institute of Applied Economic and Social Research University of Melbourne Melbourne Australia; ^4^ Centre for Mental Health Research Research School of Population Health The Australian National University Canberra Australia

**Keywords:** online, Internet, recruitment, feasibility, representativeness, postpartum

## Abstract

**Background:**

Online recruitment is feasible, low-cost, and can provide high-quality epidemiological data. However, little is known about the feasibility of recruiting postpartum women online, or sample representativeness.

**Objective:**

The current study investigates the feasibility of recruiting a population of postpartum women online for health research and examines sample representativeness.

**Methods:**

Two samples of postpartum women were compared: those recruited online as participants in a brief survey of new mothers (n=1083) and those recruited face-to-face as part of a nationally representative study (n=579). Sociodemographic, general health, and mental health characteristics were compared between the two samples.

**Results:**

Obtaining a sample of postpartum women online for health research was highly efficient and low-cost. The online sample over-represented those who were younger (aged 25-29 years), were in a de facto relationship, had higher levels of education, spoke only English at home, and were first-time mothers. Members of the online sample were significantly more likely to have poor self-rated health and poor mental health than the nationally representative sample. Health differences remained after adjusting for sociodemographic differences.

**Conclusions:**

Potential exists for feasible and low-cost e-epidemiological research with postpartum populations; however, researchers should consider the potential influence of sample nonrepresentativeness.

## Introduction

Internet-based recruitment and data collection for epidemiological research has been shown to offer benefits in terms of feasibility and accessibility (ie, low-cost, time-efficient, access to hard-to-reach populations, broad geographical/global reach) [1,2]. Importantly, evidence is also accumulating that data obtained via Internet-based research methods is reliable and valid [3-5]. Research also suggests that Internet recruitment can provide a sample of young adults broadly representative of the target population of interest [1,2,6,7].

One specific population-group in which Internet-based research and data collection may be highly enabling, but where little research is available, is postpartum women (first 12 months after birth). Previous research investigating postpartum parents has relied largely on recruitment via hospitals and child/maternal health clinics, requiring considerable time and financial investment. However, there may be significant emerging opportunities to recruit this population more efficiently online. Internet usage in this group is likely to be high, given postpartum women are often socially isolated at home, restricted in their mobility, and time-poor [8-10]. Studies successfully recruiting women planning a pregnancy (preconception) [11,12], and small samples of postpartum women [9,10], suggest that online recruitment is feasible. However, little is known about how participants recruited online might differ from postpartum women in the general population.

The current study investigates the feasibility of recruiting a large sample of postpartum women online for health research, and examines sample representativeness. Sociodemographic and health characteristics were compared between an online sample and another postpartum sample that was recruited face-to-face in the context of a nationally representative household survey.

## Methods

### Participants and Procedure

#### The Living with a Young Baby Survey

Participants were recruited during January and February 2015 for an anonymous online survey investigating women’s general postpartum health and psychological wellbeing. Online recruitment involved two processes: (1) advertisement on a popular Australian pregnancy/infant-focused website *babycentre* [13], and (2) targeted Facebook advertisements. The brief advertisements targeted women who were aged >18 years, who resided in Australia with a young baby: “ *Research: Mums Wanted! Researchers are looking for Australian women with a young baby to do a brief survey* ”. No incentive was provided for participation. Mental health was not mentioned, in an effort to minimize health selection. Individuals who *clicked* on the advertisement were redirected to the online survey, which took approximately 15 minutes to complete. The Living with a Young Baby Survey (LYBS) was completed by 1083 respondents. The LYBS was approved by The Australian National University Human Research Ethics Committee.

#### Household Income Labour Dynamics in Australia Study

Comparative data were drawn from waves (time-points) 11 and 13 of the Household Income Labour Dynamics in Australia Study (HILDA), as at these time-points the K-10 Psychological Distress Scale was included in the survey. HILDA is a longitudinal nationally representative household panel survey that has been conducted annually since 2001 [14]. At baseline, the study recruited participants using a multi-stage sampling approach, sampling households within a selection of administrative areas. At baseline, 7682 households were involved, including 13,969 individual household members aged 15-95 years. Completion of the HILDA survey involves both a brief face-to-face personal interview and a paper-pencil self-completed questionnaire. The baseline response rate (66%) and individual-level reinterview rates (96% in wave 13) are comparable or superior to other national household panels around the world [15]. The sample is not static, as new participants enter study households and a significant top-up sample was introduced in wave 11. In addition, weights provided with the dataset enable adjustment for selection and attrition to better reflect the national population [16]. The current HILDA analyses were restricted to postpartum women aged 18-50 years (youngest child <1 year), providing a total of 579 respondents (wave 11 n=288; wave 13 n=291).

### Measures

*Sociodemographic* measures included location (state and remoteness), language spoken at home, age, relationship status, number of children, and level of education completed. *General self-rated health* was measured by asking, “In general, how would you say your own health is?” Possible responses were, “excellent”, “very good”, “good”, “fair”, or “poor” [17]. *Psychological distress* was measured using the K-10 Kessler Psychological Distress Scale [18]. The K-10 has ten items representing symptoms of psychological distress, with a total scale score ranging from 10-50. The total score was categorized into four groups: 10-15 (*Low*), 16-21 (*Moderate*), 22-29 (*High*), and 30-50 (*Very High*), consistent with previous research [19,20].

### Statistical Analyses

Descriptive statistics for sociodemographic and health variables from both samples are presented. The data reported for the HILDA sample was weighted by the cross-sectional population weights included in waves 11 and 13. Chi-square tests of association identified characteristics that differed between the LYBS and HILDA samples. Univariate logistic regression provided estimates of the magnitude of differences in characteristics between the LYBS and HILDA samples (odds ratios). Multivariate logistic regression (including all variables) tested whether differences in sociodemographic characteristics accounted for differences in general health and mental health between the samples. The weighted HILDA data provides the best possible comparison group available: postpartum women can be identified, self-rated health and the K-10 are reported (comparable measures to the LYBS), and the sample is representative of the Australian population.

## Results

Online recruitment from the *babycentre* website took place over 9 days, and 264 surveys were completed. There was no cost associated with advertising on the *babycentre* website. Online recruitment from Facebook took place over 4 days, and 819 surveys were completed. Information provided by the Facebook Ads Manager shows that across the 4 days the advertisement was *clicked on* 2647 times, and the total audience reach was 38,765. Given that 819 surveys were completed, we estimate a conversion rate of *click*-to-completed-survey of 30.94% (819/2647). The total cost for advertising on Facebook was Aus $448.68 (the cost per *click* on the advertisement was Aus $0.17), and cost per survey completion was Aus $0.55.

Multimedia Appendix 1 presents both descriptive information and results from logistic regression models. Compared to mothers in the nationally representative HILDA survey, mothers in the online LYBS were more likely to be located in the Australian Capital Territory (+4.9%), to be located in a remote or rural area (+1.5%), to speak English only at home (+15.4%), to be younger (+7.7%), to be in a de facto relationship (+7.4%), and to be a first-time mother (+16.5%). Mothers in the online survey were less likely to have *not completed* high school (-5.5%). Mothers in the online LYBS had poorer general health and higher psychological distress than those in the HILDA study. Multivariate logistic regression (final column in Multimedia Appendix 1) showed that including all sociodemographic characteristics did little to attenuate the association between poor general health (and poor mental health) and participation in the LYBS online sample (compared to the HILDA sample). Those with fair/poor general health (as opposed to excellent health) remained 2.66 times more likely to participate in the LYBS online sample, compared to the HILDA sample. Those with moderate distress (as opposed to low distress) remained 2.73 time more likely to participate in the LYBS sample; those with high distress were 2.03 times more likely, and those with very high distress were 3.92 times more likely.

## Discussion

The online recruitment strategy was highly time-efficient and low-cost. Over a period of thirteen days, 1083 participants were recruited for a total direct cost of Aus $448.68. The recruitment cost for the current online study is significantly lower than has been reported previously in studies conducting online recruitment [1,21]. This result is likely due to the combined strategy of utilizing free website advertising and commercial Facebook advertising, and because the current recruitment approach targeted a very specific population (postpartum women).

There were several significant differences in sociodemographic characteristics between the online LYBS sample and the HILDA sample. One of the greatest differences was that the LYBS recruited more first-time mothers. First-time mothers may be more likely to self-select into research focused on postpartum experiences, given the experience of motherhood is new and highly salient. The higher education levels observed in the LYBS sample likely reflect greater education among social media users in general [22], and in the context of motherhood, those who engage with infant care and development websites. Women in the online LYBS sample had poorer general health and mental health. Previous research has similarly found that online recruitment for mental health surveys attracts a sample with higher levels of psychological distress than is reported by the general population [1]. This finding may be due to the fact that online recruitment is more susceptible to uncontrolled *snowballing* as participants recruit others that they think will be interested in the research. It is also possible that reports from the general population under-represent levels of psychological distress [23,24]. Given that the poorer general health and mental health of postpartum mothers recruited online was not simply due to sociodemographic variation, further investigation is needed to understand who participates in online health research and why, and how the characteristics of this population may affect the results obtained.

The extent to which sample differences associated with recruitment method will impact on the validity of research findings largely depends on the research question being investigated [5,25,26]. The current findings suggest that when the goal is to estimate the prevalence of physical and mental health problems in postpartum women, Internet recruitment will likely provide an overestimate. Alternatively, when the focus is on the relationship between exposure and illness (etiology), Hatch et al state, “representativeness of a source population is arguably not a prerequisite for either internal validity or generalizability” [5]. Nonrepresentation is chiefly a problem when characteristics that differentiate those online from those not online (eg, in this study: primiparity, non-English speaking background, education), distort or moderate the association between exposures and outcomes of interest [25].

This study recruited a large sample of postpartum mothers online, demonstrating both efficiency and low-cost. Online samples may over-represent first-time mothers with higher education and English-speaking backgrounds, as well as those experiencing physical and mental health problems. Unmeasured differences in survey methodology play a selection role (ie, length of survey, anonymity in the LYBS), and the findings may be unique to the Australian context. While the monetary figures clearly demonstrate that online recruitment is low-cost, we did not specifically conduct cost-effective analyses. Researchers adopting Internet methods to investigate postpartum health should consider the implications of sample nonrepresentation in relation to their specific research aims (eg, prevalence, etiology, intervention).

## Appendix

Multimedia Appendix 1

## Abbreviations

DSS: Department of Social Services
HILDA: Household Income Labour Dynamics in Australia Study
LYBS: Living with a Young Baby Survey
NHMRC: National Health and Medical Research Council
