# Supplementary material for: Online Recruitment: Feasibility, Cost, and Representativeness in a Study of Postpartum Women
Source: J Med Internet Res. 2017 Mar 8;19(3):e61. doi: 10.2196/jmir.5745 (PMC5362693; doi:10.2196/jmir.5745)
Supplement: Multimedia Appendix 1 [file jmir_v19i3e61_app1.pdf]

Multimedia Appendix 1. Descriptive data, and univariate and multivariate models predicting the odds of being from the online LYBS sample (versus HILDA sample).

	**Descriptive frequency data**		**Logistic Regression**	
	**LYBS (n=1083)**<br><br>**% (95% CI)**	**HILDA (n=579)**<br><br>**% (95% CI)**	**Univariate model**<br><br>**Odds Ratio (95% CI)**	**Multivariate model**<br><br>**Odds Ratio (95% CI)**[a]
State				
New South Wales	27.7 (25.0-30.4)	30.6 (26.8-34.4)	Ref	Ref
Victoria & Tasmania	22.5 (20.1-25.0)	24.2 (20.7-27.7)	1.03 (.78-1.36)	.91 (.67-1.24)
Queensland	25.3 (22.7-27.9)	23.5 (20.1-27.0)	1.19 (.90-1.57)	1.00 (.73-1.37)
South Australia & Northern Territory	6.3 (4.9-7.8)	8.1 (5.9-10.3)	.86 (.57-1.30)	.79 (.50-1.26)
Western Australia	10.5 (8.7-12.3)	10.7 (8.2-13.2)	1.08 (.75-1.55)	1.08 (.72-7.62)
Australian Capital Territory	**7.8 (6.2-9.5)**	**2.9 (1.5-4.3)**	2.99 (1.71-5.23)**	3.09 (1.70-5.64)**
Remoteness				
Major City	71.3 (68.6-74.0)	71.3 (67.6-75.0)	Ref	Ref
Inner Regional	19.0 (16.7-21.3)	19.0 (15.8-22.2)	1.0 (.77-1.30)	.91 (.67-1.22)
Outer Regional	7.1 (5.6-8.6)	8.7 (6.4-11.0)	.82 (.56-1.20)	.74 (.48-1.14)
Remote/ Very Remote	**2.5 (1.6-3.4)**	**1.0 (.2-1.8)**	2.57 (1.03-6.4)*	2.42 (.91-6.45)
English only at home				
No	**10.4 (8.6-12.2)**	**25.8 (22.2-29.4)**	Ref	Ref
Yes	**89.6 (87.8-91.4)**	**74.2 (70.6-77.8)**	2.99 (2.29-3.92)**	3.26 (2.40-4.43)**
Age categories				
18-24	16.9 (14.7-19.1)	13.3 (10.5-16.1)	Ref	Ref

25-29	**31.7 (28.9-34.5)**	**24.0 (20.5-27.5)**	1.05 (.75-1.46)	.88 (.59-1.13)
30-34	33.6 (30.8-36.4)	37.0 (33.1-40.9)	.72 (.53-.99)*	.67 (.44-1.01)
35-39	**14.3 (12.2-16.4)**	**20.6 (17.3-23.9)**	.55 (.39-.79)*	.52 (.33-.82)**
40+	3.5 (2.4-4.6)	5.0 (5.2-6.8)	.55 (.32-.96)*	.71 (.37-1.36)
Relationship status				
Married	68.2 (65.4-70.1)	71.3 (67.6-75.0)	Ref	Ref
De facto relationship	**24.2 (21.6-26.8)**	**16.8 (13.8-19.9)**	1.52 (1.17-1.97)*	1.22 (0.89-1.66)
Divorced/separated	2.8 (1.8-3.8)	3.1 (1.7-4.5)	.94 (.52-1.72)	.98 (.50-1.92)
Never married/not de facto	**4.9 (3.6-6.2)**	**8.8 (6.5-11.1)**	.58 (.39-.87)*	.38 (.23-.61)**
Number of children				
1	**60.4 (57.5-63.3)**	**43.9 (39.9-47.9)**	Ref	Ref
2	**28.0 (25.3-30.7)**	**38.1 (34.4-42.1)**	.53 (.43-.67)**	.53 (.41-.68)**
3+	**11.6 (9.7-13.5)**	**18.0 (14.9-21.1)**	.46 (.35-.63)**	.47 (.33-.66)**
Education				
Incomplete high school	**8.5 (6.8-10.2**)	**14.0 (11.2-16.8)**	Ref	Ref
Completed high school	43.6 (40.7-46.6)	41.8 (37.8-45.8)	1.71 (1.22-2.40)*	1.90 (1.29-2.81)*
Completed bachelor	47.7 (44.7-50.7)	44.2 (40.2-48.3)	1.77 (1.27-2.47)*	2.49 (1.63-3.80)**
Self-rated general health				
Excellent	**11.2 (9.3-13.1)**	**16.0 (13.0-19.0)**	Ref	Ref
Very good	41.2 (38.3-44.1)	44.8 (40.8-48.9)	1.32 (.97-1.80)	1.11 (.079-1.56)
Good	33.4 (30.6-36.2)	33.5 (29.7-37.3)	1.43 (1.04-1.97)*	1.02 (.70-1.47)
Fair/Poor	**14.2 (12.1-16.3)**	**5.9 (4.0-7.8)**	3.48 (2.20-5.52)**	2.66 (1.57-4.50)**
Psychological distress (K10)				
Low	**45.8 (42.8-48.8)**	**68.5 (64.7-72.3)**	Ref	Ref
Moderate	**29.7 (27.0-32.4)**	**18.1 (15.0-21.2)**	2.45 (1.90-3.17)**	2.73 (1.86-3.28)**

High	**14.8 (12.7-16.9)**	**9.7 (7.3-12.1)**	2.28 (1.64-.3.19)**	2.03 (1.39-2.97)**
Very high	**9.7 (7.9-11.5)**	**3.7 (2.2-5.2)**	4.05 (2.48-6.12)**	3.92 (2.26-6.78)**

Note: LYBS: Living with a Young Baby Survey. HILDA: Household Income Labour Dynamics in Australia (HILDA) Study. CI: Confidence Interval. K10: Kessler Psychological Distress Scale. SD: Standard Deviation. OR: Odds Ratio. K10: Kessler Psychological Distress Scale. Bold figures indicate significant differences between observed and expected cell values ($p<.05$) using chi-square tests (determined with an adjusted residual score <-2.00 or >2.00. [21]). COLUMNS 3 and 4: * $p<.05$; ** $p<.001$. a: Cox & Snell $R^2 = .156$.
